# Metal and oxidative potential exposure through particle inhalation and oxidative stress biomarkers: a 2-week pilot prospective study among Parisian subway workers

**DOI:** 10.1007/s00420-024-02054-2

**Published:** 2024-03-20

**Authors:** Jean-Jacques Sauvain, Maud Hemmendinger, Thomas Charreau, Valérie Jouannique, Amélie Debatisse, Guillaume Suárez, Nancy B. Hopf, Irina Guseva Canu

**Affiliations:** 1 Department of Occupational and Environmental Health, Center for Primary Care and Public Health (Unisanté), University Lausanne, Route de la Corniche 2, 1066 Epalinges, Switzerland; 2 Service Santé-Travail, Régie autonome des transports parisiens (RATP), 88 Boulevard Sébastopol, 75003 Paris, France

**Keywords:** Exhaled breath condensate, Urine, Metals, Subway, Particulate matter, Biomarkers of oxidative stress

## Abstract

**Objective:**

In this pilot study on subway workers, we explored the relationships between particle exposure and oxidative stress biomarkers in exhaled breath condensate (EBC) and urine to identify the most relevant biomarkers for a large-scale study in this field.

**Methods:**

We constructed a comprehensive occupational exposure assessment among subway workers in three distinct jobs over 10 working days, measuring daily concentrations of particulate matter (PM), their metal content and oxidative potential (OP). Individual pre- and post-shift EBC and urine samples were collected daily. Three oxidative stress biomarkers were measured in these matrices: malondialdehyde (MDA), 8-hydroxy-2′deoxyguanosine (8-OHdG) and 8-isoprostane. The association between each effect biomarker and exposure variables was estimated by multivariable multilevel mixed-effect models with and without lag times.

**Results:**

The OP was positively associated with Fe and Mn, but not associated with any effect biomarkers. Concentration changes of effect biomarkers in EBC and urine were associated with transition metals in PM (Cu and Zn) and furthermore with specific metals in EBC (Ba, Co, Cr and Mn) and in urine (Ba, Cu, Co, Mo, Ni, Ti and Zn). The direction of these associations was both metal- and time-dependent. Associations between Cu or Zn and MDA_EBC_ generally reached statistical significance after a delayed time of 12 or 24 h after exposure. Changes in metal concentrations in EBC and urine were associated with MDA and 8-OHdG concentrations the same day.

**Conclusion:**

Associations between MDA in both EBC and urine gave opposite response for subway particles containing Zn versus Cu. This diverting Zn and Cu pattern was also observed for 8-OHdG and urinary concentrations of these two metals. Overall, MDA and 8-OHdG responses were sensitive for same-day metal exposures in both matrices. We recommend MDA and 8-OHdG in large field studies to account for oxidative stress originating from metals in inhaled particulate matter.

**Supplementary Information:**

The online version contains supplementary material available at 10.1007/s00420-024-02054-2.

## Introduction

Compared to outdoor ambient air, subway environments are characterised by a higher concentration of particulate matter (PM). In addition, the subway particles are coarser and have a very different chemical composition (Loxham and Nieuwenhuijsen 2019; Seaton et al. 2005). PM sampled from underground transportation systems consists mainly of transition metals (Fe, Cu, Al, and Mn) (Ben Rayana et al. 2022; Canu et al. 2021a; Loxham and Nieuwenhuijsen 2019) with a lower content of organic compounds compared to ambient PM. These differences relate to the type of emission sources, with abrasion/mechanical wear being important in subway environments, whereas secondary aerosols and combustion emissions being the major ambient PM source (COMEAP 2018a). Extrapolation of epidemiological results relating health effects to ambient PM exposures to subway PM exposures is thus questionable. Specific epidemiological studies with subway PM exposures are, therefore, needed (COMEAP 2018b).

The complex mixture of essential or non-essential metals (Martinez-Finley et al. 2012) and their higher concentrations in subway PM compared to ambient PM could be an important determinant for explaining the observed in vitro or in vivo biological effects. In vitro studies suggest that subway PM present generally a greater oxidative potential (OP) compared to ambient PM (Janssen et al. 2014; Karlsson et al. 2005) but could be less inflammagenic (COMEAP 2018b). Indeed, the presence of transition elements in lower (Cu^+^, Mn^2+^) or mixed (Fe^2+^/Fe^3+^) oxidation states with their ability to act as electron donors or interact with important functions of proteins (e.g., sulfhydryl groups) could be responsible for the generation of reactive oxygen species (ROS). Such ROS might originate from Fenton-like reactions or result from metals interfering with the antioxidant homeostasis, inducing oxidative stress (Loxham and Nieuwenhuijsen 2019; Valko et al. 2016). Oxidative stress is an important pathway leading to pulmonary and cardiovascular diseases associated with PM exposures (Nel 2005). Metal concentrations in biological systems and particularly their soluble part need thus to be tightly controlled (Krezel and Maret 2021) through uptake, storage or elimination processes (Valko et al. 2016). By analogy to ambient particles, transition elements such as Cu, Mn, Ni or Zn are expected to be the mostly water-soluble and thus, quite bioavailable (Palleschi et al. 2018). The importance of soluble transition metals in inducing inflammatory response after exposure to particles is well described (Costa and Dreher 1997; McNeilly et al. 2004). In the case of subway PM, the soluble part of the ultrafine fraction has been shown to contribute to the in vitro cellular ROS generation (Loxham et al. 2015). Moreover, strong correlations between metals (Cu, Fe, Mn, Ni, and Zn) and the intracellular generation of ROS were also reported for cells derived from a non-small cell lung carcinoma (NCI-H727) (Spagnolo et al. 2015). Finally, the release of the chemoattractant cytokine IL-8 following an exposure of epithelial alveolar cells (A549) to subway PM has been attributed mainly to the soluble metal fraction (Seaton et al. 2005).

Very few human studies are available regarding the health effects of exposure to subway PM (reviewed in (Loxham and Nieuwenhuijsen 2019)) and no consistent acute or chronic effects has been observed from these studies. Inflammatory response through elevated plasma concentration of plasminogen activator inhibitor-1 (Bigert et al. 2008), activation of the immune system (Klepczynska Nystrom et al. 2010) or in lipid mediators (Lundstrom et al. 2011) and metabolism (Sauvain et al. 2022) in healthy volunteers following exposure to subway PM have been reported.

Based on this knowledge, we postulate that metal-rich subway PM with OP properties (Moreno et al. 2017) can increase the ROS release after deposition in the lungs followed by a perturbation of the cellular redox homeostasis. Such disturbance might result in lipid and DNA oxidation. 8-iso-prostaglandin F_2α_ (8-isoprostane) and malondialdehyde (MDA) are considered potentially relevant biomarkers of lipoperoxidation as they result from the oxidation of arachidonic acid (Basu 2008) and polyunsaturated fatty acids (Lykkesfeldt 2007), respectively. The guanine base of DNA is easily oxidised to produce the 8-hydroxyguanine. The resulting damaged DNA is repaired by eliminating the water-soluble 8-hydroxy-2′deoxyguanosine (8-OHdG), which is considered a good biomarker of oxidative stress (Graille et al. 2020b).

The aim of this pilot study was to explore the relationship between exposure to metals and OP of subway particles and the induction of an oxidative stress response in exhaled breath condensate (EBC) and urine using three effect biomarkers: MDA, 8-OHdG and 8-isoprostane. We expect subway workers to have greater PM exposures than subway commuters, and consequently, a greater chance of detecting possible biological responses. The obtained results should help in defining the most relevant biomarkers to measure in a large epidemiological study (Canu et al. 2021b).

## Material and methods

### Study design

The design of this pilot study consists of a prospective exposure and biomarker monitoring study conducted among a small group of non-smoking workers. Nine subway workers (three station agents, three locomotive operators, three security guards) from the Parisian urban transport company (RATP) in France were recruited and followed over their entire work shift (about 6 h). Among the station agents, two worked at the ticket information and sales counter, and one was a mobile station agent. The latter was responsible for supervising the purchase of tickets and providing information to customers from the ticket office he occupied on an underground metro line (line 7). Locomotive operators were making several journeys between termini on the same underground line 7. They stayed the majority of their work shift inside the train cabin. Security guards were responsible for maintaining order at the various stations, as well as on board of the metro, trains, bus and tram lines they use to travel around the network. Field measurements were collected over 6 weeks between 7th of October to 15th of November 2019 according to a registered research protocol (Canu et al. 2021b). All workers provided informed written consent prior to enrolment and completed an epidemiological questionnaire. The latter was used for collecting the participants’ sociodemographic and lifestyle-related data and potential confounding and effect-modifying variables for the exposure–response modelling.

### Personal air and biological monitoring

The personal PM_10_ and PM_2.5_ measurements were conducted for the full 6-h shifts over two consecutive weeks. At RATP, occupational hygiene measurements for air pollutants are collected by a technician that follows the worker selected for monitoring throughout the shift. A rotation for monitoring locomotive operators was set up (one driver monitored every three days), with the aim of exhaustively covering individual differences in each agent's driving habits. Time-weighted average mass concentrations expressed as PM_10_ or PM_2.5_ and their respective metal concentration (Al, As, Ba, Cd, Cr, Cu, Fe, Mn, Ni, Pb, Zn) were determined for each workday following standard procedures described in more detail in Canu et al. (2021a). The measurement of the OP of the particulate (PM_10_) and gaseous fraction was based on the ferrous xylenol orange (FOX) assay. The kinetic of the Fe^2+^ oxidation to Fe^3+^ was followed colorimetrically, taking advantage of a multiscattering absorption enhancement principle to reach low detection levels (Goekce et al. 2022). OP^FOX^ measurements were collected with a sampling train composed of a Teflon glass fibre filter connected to a XAD-2 sorbent tubes (Sauvain et al. 2021). A total of 28 filters and 31 XAD-2 sorbent tubes in addition to 18 field blanks were collected. All OP^FOX^ results have been blank corrected and expressed as pmol H_2_O_2 equivalent_/m^3^. For each series of OP^FOX^ measurements, a calibration with H_2_O_2_ standard solutions (0–9.8 µM) was performed.

EBC and urine were selected because more invasive sampling methods will discourage worker participation (Creze et al. 2021). These matrices account for oxidative stress in the target organ (EBC) and systemically (urine). They were collected daily at the same time pre- and post-shift to minimize potential influence of circadian rhythm on the measured biomarkers (Kanabrocki et al. 2002; Sani et al. 2015). EBC samples were collected during a 20 min calm oral respiratory ventilation using a portable collection device (Turbo-DECCS, Medivac, Parma, Italy) set at − 10 °C. None of the participants declared drinking coffee within the hour before EBC collection. Urine samples were collected in 120 ml plastic bottles, previously washed with nitric acid (10%) and distributed to workers the day before collection. The biological samples (EBC, 2–3 ml, urine < 100 ml) were aliquoted immediately upon reception and remotely from the sampling area on a clean table to avoid sample contamination. Collected aliquots were frozen at − 20 °C, transported, and stored either at − 80 ^◦^C (EBC) or at − 20 °C (urine) until analysis (Canu et al. 2021a).

### Quantification of metals and biomarkers of oxidative stress

The quantification of the PM metal content was achieved following an accredited method using a two-step digestion procedure (HCl 30% during 30 min at 95 °C then HNO_3_ 65% during 15 min at 95 °C). The 11 selected elements were quantified using inductively coupled plasma mass spectroscopy. The metal content in EBC was determined by aspirating a sample of 500 µL EBC acidified with 50 µL HNO_3_ 3% containing 100 µg/L Yttrium as internal standard in an ICP-MS (Thermo iCAP TQ, ThermoScientific) using external calibration for quantitation. Additional information about the analytical method is available in the Supplementary material Table S1. For urine, 250 µL of sample was diluted with 4750 µL HNO_3_ 3% containing 100 µg/L Yttrium as internal standard. Validated LC–MS methods were developed for the simultaneous analysis of 8-OHdG and 8-isoprostane in EBC (Hemmendinger et al. 2022) and urine (Sambiagio et al. 2021). MDA analysis in EBC (MDA_EBC_) was performed according to Hemmendinger et al. (2021). We used a headspace-GC–MS method described by Shin et al. (Shin and Jung 2009) for MDA analysis in urine (MDA_urine_). The limit of detection and other analytical parameters for these methods are presented in Supplementary material Table S2.

### Data management and statistical analysis

#### Data processing

Individual data from questionnaires were digitized and double-checked for correctness and completeness. The distribution of continuous quantitative variables corresponding to the measured OP, metal or biomarker concentrations was first examined graphically. All the variables were then log-transformed for normalization.

#### Modelling of censured data and descriptive statistics

A proportion of the measurements for some elements and biomarkers in both matrices fell below the limit of detection (LOD) or in the interval between LOD and limit of quantification (LOQ, see Supplementary material, Table S1 and S2). To minimize bias arising from censored data, we excluded from the analysis metals with more than 50% of measurements below the LOQ. We thus considered only Al, Fe and Zn in PM_2.5_ and Al, Cu, Fe, Mn and Zn in PM_10_. On the contrary, most of the selected metallic elements in EBC or urine could be appropriately quantified in these two matrices. For effect biomarkers, we used the interval regression, which takes into account the censured values while estimating the daily average concentration of each biomarker (Gelman and Hill 2006). For predictor variables (i.e., exposure components like metals and OP) with less than 50% of measurements below LOQ, we replaced the values below LOD and between LOD and LOQ by the centered value of the interval, respectively LOD/2 and (LOD + LOQ)/2 (Ortega-Villa et al. 2021). Then, the daily average for each exposure variable was calculated for each participant. Statistical analyses were not stratified by occupation, as no hypotheses were formulated regarding the effect of occupation on the level of the biomarkers. We considered that these concentrations were sufficiently homogeneous to allow a robust statistical analysis with an adequate number of measurements. To explore the potential latency time between exposure and oxidative stress response, we also generated lagged exposure variables, by considering exposure concentrations measures 0, 12 and 24 h prior to the oxidative biomarker measurement.

#### Exploratory correlation analysis and data visualization for metals

The relationship between metals in PM, EBC and urine was described previously (Canu et al. 2021a). In this study, we first explored relations of potential interest between different metals in PM, EBC and urine by visualizing a heat-map based on pairwise Pearson correlation (Supplementary material, Figure S1). Second, we plotted the time-series of urinary metal concentrations and urinary biomarker concentrations for all pairs of metals and biomarkers to determine which sampling time (pre-, post-, or between-shift difference) would better reflect the measured exposure and biomarker concentrations (Supplementary material, Figure S2).

#### Multivariate analysis of oxidative stress biomarkers and relationship with metals

For the effect biomarkers pre-identified during the exploratory phase as correlated with exposure variables, we performed regression analysis. In human observational studies, such as ours, individual-related variables between measured exposure and outcomes have potential confounding factors (e.g., country of birth, ethnicity, gender, age, place and habits of life, and individual susceptibility) and can bias the study results. We included these as independent variables in a multivariable regression model to reduce bias due to individual variability (Dwivedi and Shukla 2020). We started with a multilevel mixed-effect model with only one exposure variable (i.e., daily average concentrations measured the same day as the effect biomarker (no lag)) included as an independent fixed-effect variable, the participant’s ID included as a random effect variable, and participant’s age and sex as fixed-effect covariates. Furthermore, an adjustment for micronutrient consumption was added when modelling the association with exposure to Cu, Fe and Zn. The additional adjustment for body mass index (BMI), weekday and commuting time was tested but not retained in the final model, as these variables did not improve the model fit according to the Bayesian Information Criterion (Neath and Cavanaugh 2012). The same single pollutant models were then run using 12- and 24-h lagged exposure variables.

All analyses were carried out with STATA statistical software, version 16 (STATA, College Station, TX, USA).

## Results

### Sample and exposure description

The main characteristics of study participants are described in Table 1. These workers correspond to healthy non-smokers with a normal built or slight overweight. They have been occupationally exposed to subway particles for 15 years on average.

**Table 1:** Sociodemographic and health characteristics of the study participants

Description	Number (%)
Number of participants [*n* (%)]	9 (100%)
Sex [*n* (%)] Women	3 (33.3%)
Men	6 (66.7%)
Age [in years; mean (sd)]	47.2 (9.43)
Length of employment [in years; mean (sd)]	14.7 (7.19)
General health score [1: very good to 8 very bad; mean (sd)]	2.2 (0.97)
BMI [mean (sd)]	25.5 (2.28)
Vitamin supplementation [*n* (%)]	1 (11.1%)
Home to work commuting [in minutes; mean (sd)]	60.8 (29.58)
Use of motor for commuting [*n* (%)]	2 (22.2%)
Commuting by foot [*n* (%)]	3 (33.3%)

*sd* standard deviation

Detailed results regarding workers’ exposures to PM including the ultrafine fraction and their total metal content have been presented elsewhere (Canu et al. 2021a). The geometric mean PM_2.5_ and PM_10_ concentration over the 10 days reached 120 ± 67 µg/m^3^ and 139 ± 103 µg/m^3^ respectively, corresponding to about 10 times the 2019 annual outdoor PM concentration in Paris (AirParif 2020).

The median metals concentrations in PM, EBC and urine are summarized in Table 2. PM_10_ contained up to 40% Fe and 20% Al. The metal concentration in PM_10_ and PM_2.5_ were in decreasing order: Al > Fe > Zn > Ba > Mn > Cu. Fe and Mn fractions in PM_10_ or PM_2.5_ were strongly correlated (Supplementary material, Figure S1), indicative of a similar source. All workers were exposed below the existing French occupational exposure limit. The particulate fraction presented the highest median OP^FOX^ of 166.5 (IQR = 43.5–405.3) pmol H_2_O_2eq_/m^3^ in comparison with the gaseous fraction of 0.34 (IQR = 0.20–0.87) pmol H_2_O_2eq_/m^3^. Interestingly, Fe, Mn and to a lesser extent Cu in PM_10_ were positively correlated with OP_part_^FOX^ (Supplementary material, Figure S1). Neither OP_part_^FOX^ nor OP_gaz_^FOX^ was correlated with oxidative stress biomarkers (Supplementary material, Table S3) and was thus not considered in the regression analyses.

**Table 2:** Median concentrations (25 and 75% interquartile range) of metals in PM, EBC and urine as well as of oxidative stress biomarkers in EBC and urine of study participants

	*n*^a^	PM_2.5_ (µg/m^3^)	*n*	PM_10_ (µg/m^3^)	*n*	EBC (ng/L)	*n*	Urine (µg/g creatinine)
Al	66 (100)	6.56 (3.74–10.75)	65 (100)	8.12 (4.62–13.62)	–^b^	–	–	–
Ba	–	–	–	–	142 (100)	207 (147–339)	144 (89)	0.87 (0.45–1.43)
Co	–	–	–	–	142 (89)	37 (26–48)	144 (71)	0.19 (0.11–0.47)
Cr	–	–	–	–	142 (74)	32 (15–52)	–	–
Cu	–	–	65 (52)	0.10 (0.08–0.17)	142 (100)	913 (617–1401)	144 (97)	5.22 (3.95–6.50)
Fe	66 (100)	3.39 (1.28–6.86)	65 (100)	7.18 (2.57–19.37)	–	–	–	–
Mn	–	–	65 (61)	0.09 (0.08–0.18)	142 (100)	134 (99–199)	–	–
Mo	–	–	–	–	–	–	144 (100)	21.6 (14.2–31.6)
Ni	–	–	–	–	142 (100)	243 (146–439)	144 (84)	0.79 (0.59–1.46)
Pb	–	–	–	–	142 (85)	39 (23–67)	–	–
Sb	–	–	–	–	142 (54)	21 (15–31)	–	–
Si	–	–	–	–	–	–	144 (100)	5281 (4091–7123)
Ti	–	–	–	–	–	–	144 (58)	18.9 (8.1–37.9)
Zn	65 (100)	0.41 (0.36–0.50)	64 (100)	0.51 (0.36–0.83)	142 (100)	8099 (6496–10,742)	144 (98)	211 (132–287)
*Oxidative stress biomarkers*								
MDA	–	–	–	–	163 (71)	73 (56–106)^c^	144 (100)	46.1 (35.7–66.2)
8-Isoprostane	–	–	–	–	–	–	142 (96)	0.18 (0.14–0.23)
8-OHdG	–	–	–	–	–	–	144 (99)	2.86 (2.21–3.32)

^a^Number of available measurements (% of measurements > LOQ)

^b^Not analysed

^c^Units: µg/L

### Metal concentrations in biological matrices

The average metal concentrations in EBC for all exposure groups were in decreasing order: Zn > Cu > Ni > Ba > Mn (Table 2). Fe was one of the main elements in the PM; however, the majority of the EBC samples had Fe concentrations below the limit of quantification (LOQ, set at 1 µg/L) (Canu et al. 2021a). Very weak correlations were observed between the metal content of PM and metal concentrations in EBC (Supplementary material, Figure S1). Copper and Mn in EBC were strongly and positively inter-correlated but also moderately correlated with the non-essential elements Ba and Ni (Supplementary material, Figure S1). Among the non-essential elements in EBC, Sb concentration was also moderately positively associated with Ba (Supplementary material, Figure S1).

All urinary metal concentrations were creatinine adjusted. Again, most of the urinary metal concentrations for Fe and Mn were below the LOQ (set at 16 and 1.6 µg/g creatinine respectively). Urinary metal concentrations were in decreasing order: Si > Zn > Mo > Ti > Cu (Table 2). This corresponds to a different order compared to metal concentrations in PM and EBC. Except for Fe in PM_10_ and in PM_2.5_ and urinary Cu which were moderately inverse correlated (Supplementary material, Figure S1), only very weak correlations were observed between metals in PM or EBC and urine. No strong correlation within essential or non-essential elements in urine was observed, except a positive association between urinary Ba and Ti (Supplementary material, Figure S1).

### Concentrations of oxidative stress markers in biological matrices

The median and interquartile range values for the different oxidative stress biomarkers in EBC and urine are summarized in Table 2. Two of the oxidative stress biomarkers: 8-OHdG and 8-isoprostane, were not detected in EBC (limit of detection of 0.5 ng/L and 1 ng/L respectively) (Hemmendinger et al. 2022). Consequently, only MDA_EBC_ was included in the statistical analyses. The variation in the concentrations of the oxidative stress biomarkers in EBC and urine during the two consecutive weeks are presented in the Supplementary material Figure S2.

### Relationship between metals and oxidative stress biomarkers

Results of the regression analyses based on single pollutant models (i.e., Cu concentration in PM_10_) are presented in Tables 3, 4, and 5.

**Table 3:** Regression model coefficients (*β*) and their 95% confidence interval (CI) of the single pollutant model relating MDA in exhaled breath condensate (EBC) with each element in particulate matter (PM) at different lag-time and after adjustment for potential confounders

MDA EBC	No lag					12-h lag					24-h lag				
	*β*	CI inf.		CI. sup.	*p* value	*Β*	CI inf.		CI. sup.	*p* value	*β*	CI inf.		CI. sup.	*p* value
*Metals in PM*_*2.5*_															
Al	− 0.04	− 0.20	–	0.12	0.60	− 0.12	− 0.31	–	0.06	0.18	0.02	− 0.15	–	0.19	0.84
Fe	0.03	− 0.16	–	0.22	0.75	0.12	− 0.08	−	0.32	0.24	0.15	− 0.02	–	0.33	0.09
Zn	− 0.01	− 0.36	–	0.34	0.94	0.29	− 0.06	–	0.65	0.11	**0.42**	0.11	–	0.73	**0.01**
*Metals in PM*_*10*_															
Al	− 0.07	− 0.23	–	0.09	0.39	0.00	− 0.18	–	0.17	0.96	− 0.01	− 0.17	–	0.15	0.91
Cu	− 0.09	− 0.35	–	0.18	0.52	− **0.31**	− 0.58	–	− 0.04	**0.02**	− 0.14	− 0.40	–	0.11	0.27
Fe	0.08	− 0.11	–	0.28	0.41	0.02	− 0.20	–	0.24	0.85	0.13	− 0.06	–	0.32	0.18
Mn	− 0.03	− 0.31	–	0.25	0.83	− 0.07	− 0.37	–	0.22	0.63	0.09	− 0.18	–	0.35	0.52
Zn	− 0.12	− 0.41	–	0.17	0.41	0.11	− 0.19	–	0.41	0.47	0.14	− 0.14	–	0.41	0.33
*Metals in EBC*															
Ba	**0.14**	0.03	–	0.24	**0.01**	**0.13**	0.00	–	0.26	**0.05**	− 0.03	− 0.14	–	0.09	0.62
Co	0.14	− 0.10	–	0.39	0.25	**0.27**	0.01	–	0.52	**0.04**^b^	− 0.02	− 0.25	–	0.21	0.87
Cr	**0.11**	0.00	–	0.23	**0.05**	0.01	− 0.12	–	0.14	0.88	0.00	− 0.12	–	0.11	0.93
Cu	0.14	− 0.03	–	0.32	0.11	0.15	− 0.07	–	0.37	0.18	0.01	− 0.18	–	0.20	0.91
Mn	**0.17**	0.01	–	0.34	**0.04**	0.02	− 0.17	–	0.21	0.85	− 0.05	− 0.21	–	0.11	0.55
Ni	0.04	− 0.09	–	0.17	0.56	0.08	− 0.09	–	0.24	0.36	0.03	− 0.11	–	0.17	0.63
Pb	0.01	− 0.11	–	0.14	0.82	− 0.03	− 0.17	–	0.11	0.69	− 0.03	− 0.15	–	0.10	0.68
Sb	0.06	− 0.10	–	0.22	0.46	0.07	− 0.14	–	0.29	0.49	− 0.02	− 0.21	–	0.16	0.81
Zn	0.16	− 0.01	–	0.33	0.06	0.07	− 0.14	–	0.29	0.49	0.01	− 0.17	–	0.19	0.89

All models are adjusted for age and sex; models for Cu, Fe and Zn are additionally adjusted for vitamin supplementation

^a^Value in bold are statistically significative (*p* < 0.05)

**Table 4:** Regression model coefficients (*β*) and their 95% confidence interval (CI) of the single pollutant model relating MDA in urine with each element in particulate matter (PM) at different lag-time and after adjustment for potential confounders^a^

MDA urine	No lag					12-h lag					24-h lag				
	*β*	CI inf.		CI. sup.	*p* value	*β*	CI inf.		CI. sup.	*p* value	*β*	CI inf.		CI. sup.	*p* value
*Metals in PM*_*2.5*_															
Al	0.00	− 0.16	−	0.16	0.98	0.07	− 0.08	−	0.21	0.39	0.10	− 0.07	−	0.28	0.24
Fe	− 0.04	− 0.21	−	0.13	0.64	− **0.18**	− 0.32	−	− 0.03	**0.02**^b^	− 0.16	− 0.34	−	0.01	0.06
Zn	− 0.20	− 0.56	−	0.16	0.27	− 0.26	− 0.57	−	0.05	0.10	− 0.23	− 0.60	−	0.13	0.21
*Metals in PM*_*10*_															
Al	0.09	− 0.09	−	0.26	0.32	0.07	− 0.09	−	0.23	0.38	0.07	− 0.12	−	0.26	0.45
Cu	− 0.16	− 0.45	−	0.14	0.30	0.17	− 0.10	−	0.43	0.22	**0.35**	0.05	−	0.65	**0.02**
Fe	− 0.10	− 0.27	−	0.07	0.25	− 0.01	− 0.16	−	0.14	0.85	0.06	− 0.11	−	0.23	0.52
Mn	− 0.15	− 0.39	−	0.08	0.20	− 0.20	− 0.41	−	0.01	0.06	− **0.28**	− 0.52	−	− 0.03	**0.03**
Zn	− 0.16	− 0.41	−	0.09	0.21	− 0.19	− 0.41	−	0.03	0.10	− **0.29**	− 0.54	−	− 0.04	**0.02**
*Metals in urine*															
Ba	− **0.21**	− 0.36	−	− 0.07	**< 0.001**	− 0.12	− 0.27	−	0.03	0.13	− 0.10	− 0.26	−	0.07	0.26
Co	− 0.13	− 0.28	−	0.02	0.08	− 0.09	− 0.23	−	0.06	0.23	− 0.05	− 0.20	−	0.11	0.57
Cu	− 0.13	− 0.78	−	0.53	0.70	0.15	− 0.47	−	0.77	0.63	0.35	− 0.25	−	0.95	0.25
Mo	0.04	− 0.19	−	0.27	0.72	− 0.03	− 0.30	−	0.23	0.80	− 0.07	− 0.36	−	0.21	0.61
Ni	0.10	− 0.12	−	0.32	0.37	**0.22**	0.04	−	0.41	**0.02**	**0.26**	0.06	−	0.45	**0.01**
Si	0.11	− 0.37	−	0.59	0.65	0.16	− 0.32	−	0.65	0.51	0.19	− 0.35	−	0.72	0.49
Ti	0.03	− 0.15	−	0.22	0.72	0.13	− 0.09	−	0.35	0.23	0.06	− 0.17	−	0.28	0.63
Zn	− 0.15	− 0.42	−	0.11	0.25	− 0.13	− 0.36	−	0.11	0.29	− 0.05	− 0.29	−	0.20	0.70

^a^All models are adjusted for age and sex; models for Cu, Fe and Zn are additionally adjusted for vitamin supplementation

^b^Value in bold are statistically significative (*p* < 0.05)

**Table 5:** Regression model coefficients (*β*) and their 95% confidence interval (CI) with *p* value of the single pollutant model for 8-OHdG in urine, considering each element in particulate matter (PM) at different lag-time and with identical confounder adjustment^a^

8-OHdG urine	No lag					12-h lag					24-h lag				
	*β*	CI inf.		CI. sup.	*p* value	*β*	CI inf.		CI. sup.	*p* value	*β*	CI inf.		CI. sup.	*p* value
*Metals in PM*_*2.5*_															
Al	0.00	− 0.09	−	0.09	0.99	− **0.07**	− 0.13	−	− 0.02	**0.01**^b^	− 0.08	− 0.18	−	0.01	0.07
Fe	− 0.07	− 0.19	−	0.05	0.23	− 0.04	− 0.11	−	0.04	0.33	− 0.02	− 0.13	−	0.09	0.73
Zn	− 0.08	− 0.30	−	0.13	0.46	− 0.07	− 0.19	−	0.06	0.32	0.03	− 0.16	−	0.23	0.74
*Metals in PM*_*10*_															
Al	− 0.03	− 0.13	−	0.07	0.56	− 0.01	− 0.07	−	0.06	0.80	− 0.05	− 0.15	−	0.04	0.28
Cu	− 0.04	− 0.20	−	0.12	0.64	− 0.01	− 0.12	−	0.09	0.83	− 0.04	− 0.20	−	0.11	0.59
Fe	− 0.08	− 0.19	−	0.04	0.20	− 0.04	− 0.11	−	0.04	0.33	0.01	− 0.10	−	0.12	0.89
Mn	− **0.19**	− 0.36	−	− 0.02	**0.02**	No convergence					− 0.10	− 0.27	−	0.07	0.25
Zn	− 0.11	− 0.32	−	0.09	0.28	No convergence					− 0.16	− 0.33	−	0.02	0.08
*Metals in urine*															
Ba	− 0.06	− 0.19	−	0.07	0.38	0.06	− 0.06	−	0.18	0.32	0.02	− 0.10	−	0.13	0.79
Co	− **0.19**	− 0.33	−	− 0.06	**0.00**	No convergence					No convergence				
Cu	**0.37**	0.02	−	0.73	**0.04**	− 0.12	− 0.41	−	0.18	0.43	− 0.09	− 0.37	−	0.19	0.53
Mo	− **0.12**	− 0.24	−	− 0.01	**0.04**	− 0.08	− 0.19	−	0.03	0.14	− 0.08	− 0.18	−	0.02	0.10
Ni	0.01	− 0.12	−	0.15	0.85	0.07	− 0.05	−	0.19	0.24	− 0.02	− 0.14	−	0.10	0.73
Si	0.00	− 0.25	−	0.25	0.97	0.12	− 0.08	−	0.32	0.24	0.14	− 0.05	−	0.33	0.14
Ti	0.04	− 0.08	−	0.16	0.48	**0.13**	0.04	−	0.23	**0.01**	0.08	− 0.01	−	0.17	0.07
Zn	− **0.24**	− 0.44	−	− 0.04	**0.02**	− 0.09	− 0.28	−	0.09	0.32	− 0.07	− 0.24	−	0.11	0.47

^a^All models are adjusted on age and sex; models for Cu, Fe and Zn are additionally adjusted on vitamin supplementation

^b^Value in bold are statistically significative (*p* < 0.05)

The MDA_EBC_ models yielded positive and rather large *β* coefficients for the 12 and 24-h lagged exposure to Zn in PM_2.5_ (Table 3). In addition, positive but weaker *β* coefficients were observed for this oxidative stress biomarker with Mn, Ba and Cr concentrations in EBC at lag 0 (Table 3).

MDA_urine_ was negatively associated with the metal content in PM (primarily Fe, Mn and Zn) (Table 4). These associations were observed mainly 12 and 24 h after exposure, with *β* coefficients increasing with lag-time. Ba_urine_ was the only element which was negatively associated with MDA_urine_ at lag 0. Of note, only 24-h lagged exposure to Cu in PM_10_ was positively associated with MDA_urine_. The concentration of Ni_urine_ was also positively associated with MDA_urine_ at 12 or 24-h lags.

As presented in Table 5, 8-OHdG_urine_ was negatively associated with unlagged exposure to Mn in PM_10_ and with the 12-h lagged exposure to Al in PM_2.5._ The urinary concentration of essential elements (Co, Mo, Zn) was negatively associated with 8-OHdG_urine_ (Table 5) at lag 0, whereas urinary Cu presented again an opposite pattern, with a positive association with this biomarker.

We did not observe a clear association between metallic elements in PM and 8-isoprostane_urine_ (Supplementary material Table S4). Only urinary Cu at lag 0 and Ti at a 12-h lag were associated with an increase of 8-isoprostane_urine_.

## Discussion

In this pilot study, we observed subtle changes in the concentrations of oxidative stress biomarkers (mainly MDA and 8-OHdG) in EBC and urine of workers exposed to subway particles. These positive or negative changes were associated with the concentration of transition metals in PM (Cu and Zn) as well as with metals in EBC (Ba, Co, Cr and Mn) and in urine (Ba, Cu, Co, Mo, Ni, Ti and Zn). The direction of these associations was metal- and time-dependent. The associations between Cu or Zn in PM and MDA_EBC_ generally reached statistical significance only after a delayed time of either 12 or 24 h after exposure. On the contrary, changes in metal concentrations in EBC or in urine induced rapid changes in MDA_urine_ or 8-OHdG_urine_, observed mainly within the same exposure day.

### Exposure concentrations and metals measured in the biological matrices

As described previously (Canu et al. 2021a), the PM metal concentrations presented in Table 2 are in line with other reports related to subway PM characterisation (Moreno et al. 2017; Mugica-Alvarez et al. 2012). The strong correlation observed between Fe–Mn suggests similar sources, probably originating from abrasion of rail/wheels (Font et al. 2019). In addition, these PM present variable oxidative properties as measured with the FOX assay. The FOX assay presents an important selectivity to Fe and Mn content of PM, possibly explaining the highest oxidative properties measured for the locomotive operators (median OP_part_^FOX^: 941 pmol H_2_O_2eq_/m^3^; IQR: 774–1329). Indeed, this group of workers had the highest exposure to Fe based on its fraction in PM_10_ or in PM_2.5_ (Canu et al. 2021a). Similar OP_part_^FOX^ values and associations with Fe have been measured for the particulate fraction in shops using metalworking fluids (Sauvain et al. 2021). The lack of association between OP^FOX^ and the three oxidative stress biomarkers (Supplementary material, Table S3) suggests that the Fe concentration is well controlled in EBC and urine and cannot participate to the generation of radicals through Fenton reactions (Valko et al. 2016).

Once deposited in the lung, some elements from metal-rich subway particles might dissolve in the lung lining fluid. The water solubility in decreasing order for metals are Zn (~ 65% of total Zn_PM_) > Mn (~ 25% of total Mn_PM_) > Ni (~ 15% of total Ni_PM_) > Cu (< 10% of total Cu_PM_) (Mugica-Alvarez et al. 2012). It is thus, not surprising that we detected these four metals in addition to Ba in our EBC samples. Since Cu, Mn, and Zn in EBC were inter-correlated (Supplementary material, Figure S1), we suspect that these elements result from the simultaneous partial dissolution of subway particles in the lung lining fluid. Except for Zn, whose EBC concentration was rather high, all other concentration of metal elements in EBC are similar as those reported in Ghio et al. (2018) for healthy subjects. The difficulty to detect Fe in EBC in our study could result from the low solubility of subway iron-rich PM (Loxham and Nieuwenhuijsen 2019), which could be cleared from the lung through macrophage internalization and through mucociliary clearance (Ghio et al. 2018). In addition, iron homeostasis is influenced by Zn and Cu (Ghio et al. 2020) or Mn (Aguirre and Culotta 2012), which favour the iron uptake through augmented expression of metal importer and ferrireduction (reduction of Fe^3+^ to Fe^2+^) (Deng et al. 2009; Ghio et al. 2020). There is some suggestive evidence that oxidants like superoxide are produced following increase of ferrireduction (Ghio et al. 2020). Such a process could participate to the observed increased concentration of MDA_EBC_. The delayed change in MDA_EBC_ concentration 12 or 24 h after exposure to Cu or Zn in PM suggests that the release of these elements in the lungs might take some time.

The soluble PM-associated elements are thought to be quickly (within 4–24 h) distributed systemically relative to those in an insoluble state (Wallenborn et al. 2007). The urinary metal content depends on this solubility in addition to other factors like the renal function or nutrition (Fréry et al. 2017). The urinary excretion half-lives of metals are around one day for Ni (28 h, ((ATSDR) 2023), 9.6 days for Ba-sulphate nanoparticles (Konduru et al. 2014), about 10 days for Zn (Poddalgoda et al. 2019), 129 months for Cr (Petersen et al. 2000) and several decades for Cd (Suwazono et al. 2009). Comparing these half-lives in our workers with chronic exposure to subway particles (i.e., 15 years in average) suggests that a steady state should be attained in the excretion of these metals in urine. The urinary metal concentrations measured in this pilot study are quite similar to a representative sample of the general population from Spain (Domingo-Relloso et al. 2019), except for Ba, which is about 60 times lower in our study. A relatively low metal exposure might explain the lack of clear relationship between metal content in PM and urinary metal concentrations. As occupational exposure to metals among our workers were low, the urinary metal concentrations might originate from other sources.

### Concentrations and origin of biomarkers measured in the biological matrices

The concentrations of the different oxidative stress biomarkers quantified in EBC and urine (Table 2) are consistent with reference ranges proposed in the literature (Graille et al. 2020a, b; Toto et al. 2022; Turcu et al. 2022).

This pilot project assumes that the presence of metals in subway particles (biomarker of external exposure) or in EBC or urine (biomarkers of internal exposure) might induce changes in the redox homeostasis of the lung or other organs. Such modifications would result in changes of oxidative stress biomarkers either in the target organ (the lung) or systemically. As hypothesised, positive associations were observed for MDA_EBC_ with the PM_2.5_ content of Zn as well as with Co, Mn, Ba and Cr levels in EBC. This finding suggests that ROS generation and oxidative stress processes can take place in the lungs of the exposed subway workers. Once dissolved, Cr^3+^ and Mn^2+^ cations can change their oxidation state and act as catalyst for ROS generation through Fenton-like reactions (Forti et al. 2011; Valko et al. 2016). Ba is reported to bind to sulfhydryl groups in proteins altering the redox homeostasis in favour of an oxidative stress (Elwej et al. 2016). Cu in PM_10_ is the only element whose association with MDA_EBC_ was negative. Whereas signs of oxidative stress are present in EBC, the redox homeostasis in the lungs of the study participants appears to be well maintained, because MDA_EBC_ concentrations are low compared to values reported for healthy individuals (Turcu et al. 2022). In a previous study conducted with the same participants, we observed that exposure to subway particles induced changes in the anion pattern (mainly acetate, lactate and nitrogen oxides) in EBC (Sauvain et al. 2022). We interpreted such changes as an attempt of the cell/organ to maintain redox and/or metabolic homeostasis.

We observed that MDA_urine_ and 8-OHdG_urine_ were often negatively associated with transition elements in PM or urine, with the notable exception of Cu, which presents positive coefficients (Tables 4, 5). The association between exposure to metals and oxidative stress in EBC and/or urine has seldom been observed and our results are partly in line with previous studies. Workers exposed to iron oxide nanoparticles presented increased MDA_EBC_ levels compared to controls, whereas no changes in urinary MDA, 8-isoprostane or 8-OHdG could be observed for these workers (Pelclova et al. 2016). Whereas this study on nanoparticle exposure did not measure the metals content in EBC, it suggests that evaluation of oxidative stress could be more informative using EBC than urine. Few studies reported negative associations between metal exposures and 8-OHdG_urine_. In a randomized exposure-crossover study, levels of 8-OHdG_urine_ of volunteers exposed to steel mill emissions were smaller than for the same volunteers exposed to particles originating from traffic/urban emissions (Pelletier et al. 2017). A similar conclusion was reported for New York City subway workers, who presented a lower concentration of 8-OHdG compared to office workers (Grass et al. 2010). These negative associations might have multiple reasons, as the simultaneous presence of different metals in the lung and other organs might induce complex biological responses. Non-essential metals like chromium have been reported to inhibit the expression of the enzyme glycosylase 1 (OGG1), whose function is to remove adducts from DNA (Hartwig 2013). Such inhibition could reduce the excretion of 8-OHdG_urine_. On the other hand, increased concentrations of Zn, Cu (Krezel and Maret 2021) or Ba (Elwej et al. 2016) as well as interactions between Ni and Zn (Nemec et al. 2009) upregulate the production of different proteins participating to the antioxidant response element, like metallothioneins (Krezel and Maret 2017). Metallothionein functions are related to the homeostasis of essential metal ions (Zn^2+^, Cu^2+^) through metal binding with thiol functions and as a radical scavenger when these thiols functions are oxidised (Krezel and Maret 2017). The upregulation of metallothionein has been shown following exposure of epithelial cells to the ultrafine fraction from underground railways (Loxham et al. 2020). The observed negative associations between Cu in PM_10_ and MDA_EBC_ (Table 3) and Zn in PM_10_ and MDA_urine_ (Table 4) could be in line with such a protective action of both elements. Mn is also an important cofactor for manganese-superoxide dismutase (Sheng et al. 2014) and complexes between Mn and small ligands (phosphate, lactate) were reported to act as antioxidants (Aguirre and Culotta 2012). The observed negative association between Mn in PM_10_ and the concentrations of MDA_urine_ and 8-OHdG_urine_ (Tables 4, 5) could be a sign of such antioxidant effect of Mn.

Nevertheless, the scientific literature also comprises conflicting results with positive associations between subway PM or metal exposure and urinary oxidative stress biomarkers. Short-term exposures to subway PM_1_ have been shown to correlate with increase of 8-OHdG_urine_ in male volunteers (Zhang et al. 2019). Similarly, 8-OHdG_urine_ concentrations were greater in subway workers performing activities inside tunnels compared to those who were not (Mehrdad et al. 2015). Regarding metals, volunteers living near a closed zinc smelter with the highest urinary Cd presented an increased 8-OHdG_urine_ compared to volunteers with the lowest urinary Cd (Ellis et al. 2012). Boilermakers exposed to high concentrations of PM_2.5_ residual oil fly ash (median: 440 µg/m^3^) containing elevated levels of Mn, Ni, Pb and V also presented positive associations with 8-OHdG_urine_ (Kim et al. 2004). In the general population, urinary Zn [median (IQR): 198 (102–367) µg/g creatinine] was also positively associated with 8-OHdG_urine_ and MDA_urine_ (Domingo-Relloso et al. 2019). This is in contradiction with our results. The positive association between urinary Cu and MDA and 8-OHdG observed in our pilot study is in line with results from a Chinese cohort (Xiao et al. 2018) and among subjects exposed to ambient particles (Liu et al. 2018).

Significant associations between metals in particles and MDA_EBC_ or MDA_urine_ were observed with a time latency (labelled 12 or 24 h). This lag could correspond to the time needed for metal dissolution. Once present in the biological milieu, some metals (Ba, Cr and Mn in EBC; Ba in urine) have a rapid influence on the MDA concentrations. Whereas not related directly to metal exposure, an increased concentration of MDA_EBC_ immediately and 20 h after exposure to wood smoke was reported (Barregard et al. 2008). Also, the percentage change in MDA_urine_ associated with an increase of ambient PM_2.5_ was the highest at lag 0 and gradually decreased with increasing lag day (Gong et al. 2013). Similarly for 8-OHdG_urine_, associations with metals were mostly observed on the same day (Table 5), suggestive of a fast systemic response to subway particle exposure for this biomarker. This is in line with the fast increase of this DNA oxidative stress marker (within 1 h) observed after exposure of healthy volunteers to concentrated urban PM (Liu et al. 2018) and the reported half-lives of urinary excretion for 8-OHdG (6–35 h in (Chen et al. 2020; Loft et al. 1992)). In their quasi-experimental study during the 2008 Beijing Olympics, Gong et al. reported significant associations between exposure to PM_2.5_ and excretion of 8-OHdG_urine_ from the exposure day until 72–96 h after exposure with a peak association at 24–48 h (Gong et al. 2014).

### Study limitations and strengths

As all observational studies, this pilot study has some limitations that deserve discussion. The first is the low number of workers included in the study sample. The sample size was optimized considering the research and participant burden, the extensive and costly nature of measurements performed and the feasibility of their conduct without compromising the usual work of study participants and metro operation. However, this issue was partially solved by the prospective design of the study with repeated measurements, which allowed us obtaining 144 values per oxidative stress biomarker. The continuous individual exposure measurement over the work shift and twice a day measures of the outcomes for each worker decreased the inter-individual variability, improving by this way the possibility to observe modifications of the biomarker levels in EBC and urine. To improve the robustness of the statistics, we imposed stringent constrains on the data used. Only transition elements and biomarkers with more than 50% of their values above the LOQ were considered in the regression analysis. The statistical approach chosen might raise a concern of multiple comparisons and spurious associations. We studied the effects of 11 metallic elements on 4 biomarkers; with such figures, only 2 results with a *p* value below 0.05 could be simply due to chance. Given the exploratory nature of this study and in order not to miss a possible effect (i.e., to avoid type II errors), we did not correct the main analysis for multiple testing, as recommended by Armstrong (2014). However, to minimize this issue, we limited the analysis to the most salient correlational spots based on the heat-map visualisation (Supplementary material, Figure S1). Therefore, our conclusions are based on a cautious interpretation of study results and using a triangulation between different analyses performed. In this study, we did not measure the bioavailable metal fraction from PM due to constraints of the budget. It is possible that associations with the soluble fraction instead of the total metal content would have been stronger than what we observed. This aspect deserves further investigation and should be considered in future studies.

The strength of our study lies in the fact that we considered simultaneously two different biological matrices. EBC can be considered as the biological matrix associated with the target organ, where oxidative stress potentially starts. Measurement of biomarkers of oxidative stress in urine, on the contrary, corresponds to the systemic response of the body to the initial insult resulting from the subway PM deposition in the lungs. Considering the association between different exposure components and effect biomarkers in a time-dependent way using three different lag times is another important strength of this study. Such analyses are quite unique and inform upon the possible mechanism of subway particle toxicity. Finally, a prospective design enabling this analysis is a methodologically strong feature enabling the temporality condition to be met when investigating the exposure–effect associations. The results of this study will inform the large epidemiological study protocol and allow a judicious choice of the most relevant effect biomarkers to be measured. For instance, this study motivated decision to exclude the 8-OHdG and 8-isoprostane measurement in EBC in the future study (Canu et al. 2021b) and thus limit the EBC volume and study budget considerably.

## Conclusion

In this pilot study, we explored relationships between exposures to particulate matter (metals in PM) and exposure biomarkers (metals in EBC or urine) and oxidative stress biomarkers (MDA, 8-OHdG, isoprostane in EBC and urine). Although occupational exposures were overall low in our workers, we found that oxidative stress biomarkers were associated with transition metals (Cu and Zn) in PM and metals in EBC (Ba, Co, Cr and Mn) and in urine (Ba, Cu, Co, Mo, Ni Ti and Zn). The directions of these associations were metal- and time dependent, which suggest quite complex interplay between metals in the deposited PM and the lungs. Two oxidative stress biomarkers: MDA and 8-OHdG, were sensitive to internal metal exposures at very low concentrations. Thus, MDA in EBC and urinary MDA and 8-OHdG are pertinent oxidative stress biomarkers that can be used in larger epidemiological study focusing on exposure to subway PM.

### Supplementary Information

Below is the link to the electronic supplementary material.

Supplementary file1 (DOCX 686 KB)

## Data Availability

The datasets used and/or analyzed for the current study are available from the corresponding author on reasonable request.

## Abbreviations

8-Isoprostane: 8-Iso-prostaglandin F_2__α_
8-Isoprostane_urine_: 8-Iso-prostaglandin F_2__α_ in urine
8-OHdG: 8-Hydroxy-2′deoxyguanosine
8-OHdG_urine_: 8-Hydroxy-2′deoxyguanosine in urine
BMI: Body mass index
CI_95%_: 95% Confidence interval
DNA: Deoxyribonucleic acid
EBC: Exhaled breath condensate
FOX: Ferrous orange-xylenol assay
LOD: Limit of detection
LOQ: Limit of quantification
MDA: Malondialdehyde
MDA_EBC_: Malondialdehyde in EBC
MDA_urine_: Malondialdehyde in urine
OP: Oxidative potential
PM: Particulate matter
RATP: Parisian urban transport company
ROS: Reactive oxygen species
Al: Aluminium
As: Arsenic
Ba: Barium
Cd: Cadmium
Co: Cobalt
Cr: Chromium
Cu: Copper
Fe: Iron
Mn: Manganese
Mo: Molybdenum
Ni: Nickel
Pb: Lead
Sb: Antimony
Ti: Titanium
Zn: Zinc
