# Supplementary material for: Metal and oxidative potential exposure through particle inhalation and oxidative stress biomarkers: a 2-week pilot prospective study among Parisian subway workers
Source: Int Arch Occup Environ Health. 2024 Mar 20;97(4):387–400. doi: 10.1007/s00420-024-02054-2 (PMC10999389; doi:10.1007/s00420-024-02054-2)
Supplement: Supplementary file 1 — Supplementary file1 (DOCX 686 KB) [file 420_2024_2054_MOESM1_ESM.docx]

**Supplementary material**

**Metal and oxidative potential exposure through particle inhalation and oxidative stress biomarkers: a prospective study among Parisian subway workers**

J.J. Sauvain^1*^, M. Hemmendinger^1^, T. Charreau^1^, V. Jouannique^2^, A. Debatisse^2^, G. Suárez^1^, N.B. Hopf^1^ and I. Guseva Canu^1^

Table S1: Summary of the main analytical parameters for the metal measurement in exhaled breath condensate (EBC) and urine.

| Metal | m/z | EBC | Urine | | |
| --- | --- | --- | --- | --- | --- |
|  |  | LOD [ng/L] | LOD [µg/L] | Repeatability CV^a^ (%) | Biais (%) |
| Al | 27 | 1700 | 4.2 | 7.9 | 3.3 |
| Ba | 138 | 10 | 0.13 | 3.5 | -2.7 |
| Co | 59 | 10 | 0.03 | 2.5 | 2.5 |
| Cr | 52 | 10 | 0.27 | 4.0 | 6.6 |
| Cu | 63 | 70 | 0.97 | 2.3 | 4.8 |
| Fe | 57 | 300 | 8.4 | 2.7 | 5.0 |
| Mn | 55 | 10 | 0.07 | 6.2 | 11.2 |
| Mo | 98 | 10 | 1.55 | 3.8 | -1.1 |
| Ni | 60 | 10 | 0.20 | 2.7 | 3.9 |
| Pb | 208 | 10 | 0.69 | 8.9 | -16.9 |
| Si | 28 | 7000 | 33 | 5.9 | -8.5 |
| Ti | 48 | 70 | 6.7 | 9.9 | -0.2 |
| Zn | 66 | 300 | 8.10 | 1.3 | 0.07 |

^a^: coefficient of variability

Table S2: Summary of the main analytical parameters for the biomarker’s measurement in exhaled breath condensate (EBC) and urine.

|  | Analytical technique | LOD  [ng/L] | Repeatability [%] | Recovery [%] |
| --- | --- | --- | --- | --- |
| MDA_EBC_ | DNPH derivatisation - LC-MS | 25 | <10 | 92-106 |
| MDA_urine_ | PFPH derivatisation – headspace GC-MS | 3300 | <13 | 85-90 |
| 8-OHdG_urine_ | SPE - LC-MS | 10 | <10 | 97 |
| 8-isoprostane_urine_ | SPE - LC-MS | 20 | <8 | 91 |

LOD: limit of detection DNPH: Dinitrophenyl hydrazine

PFPH: Pentafluophenylhydrazine SPE: Solid phase extraction

GC-MS: Gaz chromatography coupled with mass detection

LC-MS: Liquid chromatography coupled with mass detection

Figure S1: Correlation heat map based on pairwise Pearson correlation between metal content in subway particles, in EBC and urine as well as oxidative potential. The strength of the Pearson correlation coefficient is illustrated based on the circle size and its direction based on the color scale indicated on the right-hand side.

Figure S2: Time-series for the MDA concentrations in exhaled breath condensate and urine (A) and urinary 8-isoprostane and 8-OHdG (B), averaged for all volunteers. The blue vertical line corresponds to the week-end (two days without sampling). The x-axis shows the time of biological sampling with an alternation of pre- (circles) and post-work shift (triangles) sample collection.

Table S3: Regression model coefficients (β) and their 95% confidence interval (CI) with p-value of the single pollutant model relating the different biomarkers measured in EBC and urine with the particulate and gaseous oxidative potential (OP) at different lag-time and with confounder adjustment^a^.

|  | No lag | | | | | 12-hours lag | | | | | 24-hours lag | | | | |
| --- | --- | --- | --- | --- | --- | --- | --- | --- | --- | --- | --- | --- | --- | --- | --- |
|  | β | CI inf. |  | CI. sup. | p value | β | CI inf. |  | CI. sup. | p value | β | CI inf. |  | CI. sup. | p value |
| **MDA EBC** | | | | | | | | | | | | | | | |
| OP Particles | -0.05 | -0.27 | - | 0.18 | 0.68 | 0.00 | -0.37 | **-** | 0.38 | 0.98 | 0.21 | -0.11 | - | 0.54 | 0.20 |
| OP Gaz | -0.02 | -0.16 | - | 0.12 | 0.79 | 0.12 | -0.04 | - | 0.27 | 0.14 | 0.00 | -0.13 | - | 0.14 | 0.96 |
| **MDA urine** | | | | | | | | | | | | | | | |
| OP Particles | -0.09 | -0.33 | **-** | 0.16 | 0.49 | -0.19 | -0.54 | - | 0.16 | 0.29 | 0.00 | -0.40 | - | 0.41 | 0.98 |
| OP Gaz | 0.00 | -0.15 | - | 0.14 | 0.95 | -0.01 | -0.16 | - | 0.13 | 0.88 | -0.03 | -0.20 | - | 0.13 | 0.69 |
| **8-isoprostane urine** | | | | | | | | | | | | | | | |
| OP Particles | 0.03 | -0.10 | - | 0.15 | 0.69 | -0.09 | -0.27 | - | 0.10 | 0.37 | -0.04 | -0.22 | - | 0.15 | 0.70 |
| OP Gaz | 0.03 | -0.04 | - | 0.11 | 0.38 | 0.06 | -0.02 | - | 0.13 | 0.13 | 0.02 | -0.06 | - | 0.09 | 0.67 |
| **8-OHdG urine** | | | | | | | | | | | | | | | |
| OP Particles | -0.01 | -0.13 | - | 0.11 | 0.91 | -0.06 | -0.21 | - | 0.09 | 0.46 | -0.04 | -0.25 | - | 0.16 | 0.67 |
| OP Gaz | 0.01 | -0.06 | - | 0.08 | 0.78 | 0.02 | -0.04 | - | 0.08 | 0.53 | -0.02 | -0.11 | - | 0.06 | 0.57 |

^a^: All models are adjusted on age and sex; models for Cu, Fe and Zn are additionally adjusted on vitamin supplementation.

Table S4: Regression model coefficients (β) and their 95% confidence interval (CI) with p-value of the single pollutant model for 8-isoprostane in urine, considering each element in particulate matter (PM) at different lag-time and with identical confounder adjustment^a^.

|  | No lag | | | | | 12-hours lag | | | | | 24-hours lag | | | | |
| --- | --- | --- | --- | --- | --- | --- | --- | --- | --- | --- | --- | --- | --- | --- | --- |
|  | β | CI inf. |  | CI. sup. | p value | β | CI inf. |  | CI. sup. | p value | β | CI inf. |  | CI. sup. | p value |
| Metals in PM_2.5_ | | | | | | | | | | | | | | | |
| Al | 0.04 | -0.05 | - | 0.13 | 0.41 | 0.00 | -0.10 | - | 0.10 | 1.00 | -0.01 | -0.11 | - | 0.08 | 0.76 |
| Fe | 0.02 | -0.10 | - | 0.14 | 0.79 | -0.05 | -0.16 | - | 0.06 | 0.42 | 0.00 | -0.11 | - | 0.11 | 0.97 |
| Zn | -0.04 | -0.25 | - | 0.17 | 0.73 | 0.11 | -0.08 | - | 0.30 | 0.25 | 0.14 | -0.04 | - | 0.33 | 0.14 |
| Metals in PM_10_ | | | | | | | | | | | | | | | |
| Al | 0.04 | -0.06 | - | 0.14 | 0.39 | **0.10** | 0.00 | - | 0.19 | **0.05** | 0.06 | -0.03 | - | 0.14 | 0.22 |
| Cu | 0.04 | -0.12 | - | 0.20 | 0.63 | 0.02 | -0.13 | - | 0.18 | 0.77 | -0.09 | -0.24 | - | 0.05 | 0.22 |
| Fe | 0.02 | -0.10 | - | 0.15 | 0.70 | 0.03 | -0.08 | - | 0.15 | 0.60 | 0.00 | -0.11 | - | 0.10 | 0.94 |
| Mn | -0.09 | -0.27 | - | 0.10 | 0.37 | -0.05 | -0.22 | - | 0.12 | 0.58 | -0.01 | -0.18 | - | 0.15 | 0.89 |
| Zn | 0.07 | -0.12 | - | 0.25 | 0.48 | -0.08 | -0.25 | - | 0.10 | 0.39 | -0.05 | -0.21 | - | 0.11 | 0.56 |
| Metals in urine | | | | | | | | | | | | | | | |
| Ba | -0.04 | -0.20 | - | 0.11 | 0.56 | 0.13 | -0.03 | - | 0.28 | 0.11 | 0.10 | -0.04 | - | 0.25 | 0.16 |
| Co | -0.04 | -0.23 | - | 0.14 | 0.64 | -0.03 | -0.21 | - | 0.15 | 0.75 | 0.10 | -0.09 | - | 0.30 | 0.29 |
| Cu | **0.46** | 0.05 | - | 0.87 | **0.03** | -0.26 | -0.69 | - | 0.17 | 0.23 | -0.19 | -0.59 | - | 0.20 | 0.34 |
| Mo | -0.04 | -0.18 | - | 0.10 | 0.59 | -0.08 | -0.24 | - | 0.08 | 0.35 | -0.04 | -0.18 | - | 0.11 | 0.62 |
| Ni | -0.11 | -0.27 | - | 0.05 | 0.19 | -0.06 | -0.23 | - | 0.12 | 0.51 | 0.00 | -0.16 | - | 0.17 | 0.97 |
| Si | -0.08 | -0.36 | - | 0.21 | 0.60 | 0.07 | -0.22 | - | 0.36 | 0.63 | 0.01 | -0.25 | - | 0.28 | 0.94 |
| Ti | 0.02 | -0.12 | - | 0.16 | 0.78 | **0.20** | 0.07 | - | 0.33 | **0.00** | 0.12 | -0.01 | - | 0.24 | 0.07 |
| Zn | -0.17 | -0.43 | - | 0.09 | 0.21 | -0.11 | -0.35 | - | 0.14 | 0.39 | -0.12 | -0.36 | - | 0.12 | 0.32 |

^a^: All models are adjusted on age and sex; models for Cu, Fe and Zn are additionally adjusted on vitamin supplementation.
